# Dangerous trends in pet obesity

**DOI:** 10.1136/vr.k2

**Published:** 2018-01-05

**Authors:** Alexander J. German, Georgiana R. T. Woods, Shelley L. Holden, Louise Brennan, Caroline Burke

**Affiliations:** af1 University of Liverpool, Leahurst Campus, Chester High Road, Neston CH64 7TE; af2 Crown Pet Foods, Oak Tree Meadow, Blackworthy Road, Castle Cary, Somerset BA7 7PH

Obesity is a condition in which an excess body fat has developed to the point that health is adversely affected.^1^ Dogs that are overweight have a shortened life span,^2^ their quality of life is adversely affected,^3^ and they are predisposed to other conditions including osteoarthritis, diabetes mellitus and certain types of neoplasia.^4^

At a recent World Small Animal Veterinary Association One Health meeting, canine obesity was officially classified as a disease, which is consistent with its classification in people.^5^

The last study to report obesity prevalence in pet dogs in the UK was published in 2010. The study found 59 per cent of dogs were classified as overweight or obese.^6^

Between June 2016 and October 2017, dog owners attending seven different family pet shows in five UK locations (Berkshire, Cheshire, Hertfordshire, Kent and Manchester) consented to their dog having a body condition score assessment by a team of experienced veterinary nurses. Data from 1100 adult (≥24 months) and 516 juvenile (<24 months) dogs were available for analysis. In adult dogs, 715 (65 per cent) were overweight (body condition score of 6/9 to 9/9) and 99 (9 per cent) were obese (body condition score of 8/9 or 9/9). Most concerning was the prevalence of obesity in the juvenile dogs examined, where 190 (37 per cent) and 16 (3 per cent) were classified as overweight and obese, respectively. Further, the prevalence increased steadily during the growth phase, from 21 per cent (21/100) in dogs younger than six months of age to 52 per cent (16/31) in dogs 18 to 24 months of age.

To date, the veterinary profession has not taken the problem of obesity seriously enough. For example, veterinary surgeons infrequently record weight and body condition during veterinary consultations,^7^ and rarely record the fact that a dog is overweight or obese in their clinical notes.^8^ In our opinion, veterinary professionals can help to reverse the current trend by focusing on prevention, while continuing to dedicate their time to successfully managing obesity when already developed.^9^

Veterinary surgeons infrequently record weight and body condition during veterinary consultations

Proactive monitoring of body weight and body condition throughout life would be fundamental to any such preventive plan.^10^ Given the prevalence of being overweight in growing dogs, body weight monitoring should start at initial vaccinations and continue throughout the early life phase. Such an approach is facilitated by the availability of evidence-based growth charts, for example, those recently produced for dogs weighing up to 40 kg,^11^ and freely available for use by veterinary professionals at www.waltham.com/resources/puppy-growth-charts.

## References

[1] KOPELMAN PG Obesity as a medical problem. Nature 2000:404;635–43 10766250 10.1038/35007508

[2] KEALY RD, LAWLER DF, BALLAM JM, et al Effects of diet restriction on life span and age-related changes in dogs. J Am Vet Med Assoc 2002:220;1315–20 11991408 10.2460/javma.2002.220.1315

[3] GERMAN AJ, HOLDEN SL, WISEMAN-ORR ML et al Quality of life is reduced in obese dogs but improves after successful weight loss. Vet J 2012:192;428–34 22075257 10.1016/j.tvjl.2011.09.015

[4] LUND EM, ARMSTRONG PJ, KIRK CA & KLAUSNER JS Prevalence and risk factors for obesity in adult dogs from private US veterinary practices. Int J Appl Res Vet Med 2006:4;177–86

[5] DAY MJ One Health approach to preventing obesity in people and their pets. J Comp Pathol 2017:156;293–5 28457487 10.1016/j.jcpa.2017.03.009

[6] COURCIER EC, THOMPSON RM & MELLOR DJ An epidemiological study of environmental factors associated with canine obesity. J Small Anim Pract 2010;51:362–7 20402841 10.1111/j.1748-5827.2010.00933.x

[7] GERMAN AJ & MORGAN LE How often do veterinarians assess the bodyweight and body condition of dogs? Vet Rec 2008:163;503–5 18953073 10.1136/vr.163.17.503

[8] ROLPH NC, NOBLE PJM & GERMAN AJ How often do primary care veterinarians record the overweight status of dogs? J Nutr Sci 2014:3;e58 26101626 10.1017/jns.2014.42 PMC4473162

[9] GERMAN AJ Outcomes of weight management in obese pet what: dogs can we do better? Proc Nutr Soc 2016:75;398–404 27132635 10.1017/S0029665116000185

[10] GERMAN AJ Obesity prevention and weight management after loss. Vet Clin N Amer 2016:46;913–929 10.1016/j.cvsm.2016.04.011 27255281

[11] SALT C, MORRIS PJ, GERMAN AJ et al Growth standard charts for monitoring bodyweight in dogs of different sizes. PLoS ONE 2017:12;e0182064 28873413 10.1371/journal.pone.0182064 PMC5584974

